# 

**Published:** 1917-01-06

**Authors:** 


### CONTENTS.

| | PAGE |
|---|---|
| **Professional Prospects and Anticipations** ... | 275 |
| **Immoralities in Church Management** ... ... | 276 |
| **Hospital and Institutional News** ... ... ... | 277 |
| The Canadian Medical Service—The Segregation Scheme Denounced—The V.A.D. Hospitals Justified—At the Close of the War—Wanted, a House and Grounds, in or near London, for the Red Cross—Some Outstanding Bequests of the Week—Two Medical Veterans of Distinction—National Relief Grants to Hospitals—The Hygiene of Prisoners' Camps—A Remarkable Hospital Calendar—A Base Hospital at Staincliffe Institution?—Bargaining for Hospital Tickets—The late Mr. T. Fenwick Harrison—The Welsh Medical School—Control of War Charities—A Step Retraced—London Hospital Saturday Results—A Suggestion for the Board of the King Edward VII. Hospital, Cardiff—The Function of Sanatoria—This Week's Drug Market—To Our Readers. | |
| **Surgeons, Hospitals, and Patients** ... ... | 281 |
| The Road to Standardisation. | |
| **The Institutional Library** ... ... ... ... ... | 283 |
| Arboreal Man—Home Care of Consumptives—Handbook for Wives and Mothers in India—Clinical Methods—Crowley's Hygiene of School Life — Hospital Days — With the Russian Wounded. | |
| **The Nation and its Nurses**... ... ... ... ... | 285 |
| Their Enemies' Tactics are Identical. | |
| **Round the Hospitals** ... ... ... ... ... | 286 |
| 30,000 Nurses for the College Register—Registered College Nurses to Meet Colonels and Captains for Nurses' Army—1,000 *Nursing Mirror* Readers Join College—The Nurse as Recruiting Sergeant. | |
| **Christmas in the Hospitals**... ... ... ... | 287 |
| St. Bartholomew's Hospital—Manchester Royal Infirmary—Leeds General Infirmary—Royal Infirmary, Bradford—The Royal Victoria Infirmary, Newcastle-on-Tyne—Norfolk and Norwich Hospital—County Hospital, Lincoln—General Hospital, Wolverhampton—Lord Mayor Treloar Crippled Children's Hospital, Alton, Hants—Entertainments at the Leicester Institutions. | |
| **Matrons' and Sisters' Appointments** ... ... | 292 |
| **Hospital and Institutional Results in 1915** ... | 292 |
| **Parliament and the Profession** ... ... ... | 292 |
| **The Institutional Worker** ... ... (Suppl.) | 1 |
| Precautions in Operation Cases: Answers to December Question. | |
| Questions for January. | |
| Questions Invited. | |
| Enquiries and Answers. | |

